# Exploring the Nutraceutical Potential of Dried Pepper *Capsicum annuum* L. on Market from Altino in Abruzzo Region

**DOI:** 10.3390/antiox9050400

**Published:** 2020-05-08

**Authors:** Alice Della Valle, Marilisa Pia Dimmito, Gokhan Zengin, Stefano Pieretti, Adriano Mollica, Marcello Locatelli, Angelo Cichelli, Ettore Novellino, Gunes Ak, Serife Yerlikaya, Mehmet Cengiz Baloglu, Yasemin Celik Altunoglu, Azzurra Stefanucci

**Affiliations:** 1 Department of Pharmacy, University of Chieti-Pescara “G. d’Annunzio”, Via dei Vestini 31, 66100 Chieti, Italy; alice.dellavalle@unich.it (A.D.V.); marilisa.dimmito@unich.it (M.P.D.); adriano.mollica@unich.it (A.M.); marcello.locatelli@unich.it (M.L.); 2 Department of Biology, Science Faculty, Selcuk University, 42005 Konya, Turkey; gokhanzengin@selcuk.edu.tr (G.Z.); akguneselcuk@gmail.com (G.A.); 3 National Center for Drug Research and Evaluation, Italian National Institute of Health, Viale Regina Elena 299, 00161 Rome, Italy; stefano.pieretti@iss.it; 4 Department of Medical, Oral and Biotechnological Sciences, “G. d’Annunzio” University Chieti-Pescara, Via dei Vestini 31, 66100 Chieti, Italy; angelo.cichelli@unich.it; 5 Department of Pharmacy, University of Naples “Federico II”, Via D. Montesano 49, 80131 Naples, Italy; ettore.novellino@unina.it; 6 Department of Genetics and Bioengineering, Faculty of Engineering and Architecture, Kastamonu University, 3700 Kastamonu, Turkey; serifeyerlikaya@gmail.com (S.Y.); mcbaloglu@gmail.com (M.C.B.); yasemincelikbio@gmail.com (Y.C.A.); 7 Agronomy Department, University of Florida—IFAS, 32611, Gainesville, FL 352, USA

**Keywords:** *Capsicum annuum*, nutraceuticals, chemical composition, functional ingredients

## Abstract

Sweet pepper is a typical type of *Capsicum annuum* from Abruzzo region, recognized as a traditional and local product, traditionally cultivated in the town of Altino (Abruzzo region, Italy). The aim of this study is to compare the sweet type of peppers from Altino with the hot pepper cultivated in the same area, in order to delineate their different phytochemical and biological profiles in vitro and in vivo. In this study, we elucidated their phytochemical composition, fatty acids composition and phenolic/flavonoid contents in extracts. Then antioxidant and enzyme inhibition assays were performed to evaluate their biological properties, together with in vitro cell assay and in vivo anti-inflammatory activity. Microwave (1000 mg/mL) extract of hot pepper showed the best inhibition value on in vitro cell growth assay; in fact, the number of survived cells was about 20% and 40% for microwave and Soxhlet extracts, respectively. In vivo anti-inflammatory assay revealed good activity for both species, which, when associated with in vitro cell inhibition results, could explain the protective effect on human prostatic hyperplasia.

## 1. Introduction

Peppers are vegetables belonging to *Capsicum annuum,* the most economically important genus of the Solanaceae family from South America [1]. The peppers are herbaceous or suffruticose plants, high, from 30 to 200 cm with whitish flowers, rarely purplish. The fruit is a berry with many seeds that are variable in shape, size and color. Sweet peppers can present as square or rounded fruits while spicy varieties exhibit elongated and conical fruits. The presence of capsaicin found in chili peppers confers in them a characteristic pungent taste. This alkaloid interacts with the transient receptor potential vanilloid-1 (TVR1), which is involved in the neural response to temperature variations, acidosis, pain and osmolarity [2,3]. This mechanism is responsible for heat and pungent sensation, pain relief, gastrointestinal protection and antioxidant properties [4,5]. Peppers are cultivated in the southern regions of Italy, Piedmont and Lombardy, and they are abundant and tasty during the summer season and early autumn. Peppers are eaten dried, fresh or preserved in brine or vinegar; the spicy ones are used as sauces and powder (e.g., paprika). The pepper is a source of vitamins C and E, α-carotene, β-carotene, lycopene, lutein, zeaxanthin and cryptoxanthin; at least two-thirds of human intake of these nutrients are from tomato and sweet pepper, where the latter provides 12% of the total zeaxanthin and 7% of the total consumption of vitamin C [6]. Antioxidant benefits of sweet peppers are demonstrated on human health, involving studies on the prevention of cardiovascular disease and type II diabetes [7].

Daily consumption of antioxidant nutrients can decrease cellular oxidative stress, while anti-inflammatory nutrients can reduce the insurgence of chronic inflammation [8]. Peppers are a rich supply of phytonutrients, endowed with antioxidant and anti-inflammatory properties. Here we studied the antioxidant, cell-protective and anti-inflammatory effects of two types of *Capsicum annuum*: sweet pepper and hot pepper cultivated in Altino town (*Capsicum annuum* L. var. Longum and *Capsicuum annuum* var. Acuminatum, respectively, as previously reported by Colavita et al., 2014.) [9]. 

This morphological typology is typical of Abruzzo region, locally called “Paesanello di Altino” or “A cocce capammonte”. This product is included in the traditional list of local products. The sweet pepper from Altino is peculiar to the area between the Sangro and Aventino rivers, around Altino, Roccascalegna, Bomba, Casoli, Archi, Atessa and Sant’Eusanio del Sangro in the province of Chieti. The book “Origin and history of plants cultivated in Abruzzo” by Aurelio Manzi compares an historical citation dated 1752, referring to a deed of sale in which the plant is cited as “peparol” [10].

The aim of this study is to compare the two types of peppers from Altino in order to define their different phytochemical and biological profiles in vitro and in vivo. In this work, four types of extracts were prepared and analyzed by means of HPLC-DAD to determine polyphenol and flavonoid content. Antioxidant assays involving 2,2-Diphenyl-1-picrylhydrazyl (DPPH) and 2,2ʹ-azino-bis(3-ethylbenzothiazoline-6-sulfonic acid) (ABTS), cupric reducing antioxidant capacity (CUPRAC), ferric reducing antioxidant power (FRAP), phosphomolybdenum and chelating activity assays together with a series of enzyme inhibition tests were performed to reveal antioxidant and in vitro inhibitory activities. The activity of each extract was also tested against tyrosinase enzyme which is deeply involved in the development of melanoma and inflammatory processes [11]; consequently, the inhibition of prostate cancer cell growth was also assessed. To the best of our knowledge, this is the first report focused on the study of different extracts of sweet and hot peppers from Altino. Given the potential antioxidant and anticancer properties of the pepper components, we expect to delineate an interesting panel of biological properties for these two types of local peppers from Abruzzo region. 

## 2. Materials and Methods

### 2.1. Reagents and Standards 

Analytical standards for HPLC analysis and antioxidant and enzymatic inhibitor activities were acquired from Sigma-Aldrich (Milan, Italy). The stock standard solutions were prepared following the procedure previously described by our research group [12,13]. 

### 2.2. Plant Material 

**Figure 1:**
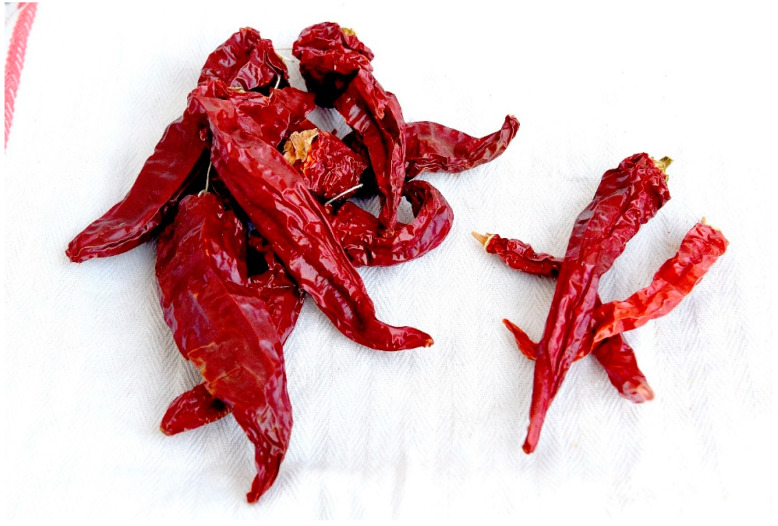
Sweet (left) and hot peppers (right) from Altino (Chieti, Abruzzo, Italy).

Whole sundried hot and sweet peppers from Altino (42°06’ N, 14°20’ E southern Italy, Chieti, Italy) (Figure 1) were purchased from the local market of Altino town in June 2019. The type along with the geographic origin were confirmed by the Associazione Produttori Peperone di Altino (Peppers Producers association of Altino), Chieti, Italy. After separation of the seeds, the material was powdered with a blender, frozen in liquid N_2_, minced and freeze-dried with a lyophilizator (VirTis, Gardiner, NY, USA). The raw material was ground to a fine powder to obtain a 40-mesh granulometry. The powder was stored in a dark vacuum box at 4 °C.

### 2.3. Extractions for HPLC-DAD Analysis

Extractions of *Capsicum annuum* powder were made by four techniques: Soxhlet extraction, microwave extraction, decoction by 60% MeOH/H_2_O solution and decoction with *n*-hexane. The extracts were lyophilized and then submitted to HPLC analysis and enzymatic inhibition assay. 

### 2.4. Sample Preparation for GC-MS Analysis

Pepper powder (100 g) was minced and suspended in distilled water; then light petroleum ether (200 mL) was added, stirring vigorously for 8 h. The suspension was filtered off and the liquid was centrifuged. The collected oil phases were dried under N_2_ flux and analyzed by GC-MS.

### 2.5. Soxhlet Extraction of Hot and Sweet Pepper Powders 

Dried powder (500 mg) was transferred into a paper filter inside the Soxhlet apparatus. Then, 60% MeOH in water solution (50 mL) was added and the system was allowed to reflux for 6 h following three extraction cycles. Finally, the solution was filtered and freeze-dried.

### 2.6. Microwave (MW) Extraction of Hot and Sweet Pepper Powders

Raw material (500 mg) was suspended in 20 mL of 60% MeOH in water in a glass, sealed vial using an automatic Biotage Initiator™ 2.0 (Uppsala, Sweden; 2.45 GHz high-frequency microwaves; power range: 0–300 W). Settings were as follows: temperature thermostatically kept at 80 °C; power: 60 W; extraction time: 5 min. The resulting suspension was filtered and freeze-dried.

### 2.7. Decoction in Methanol 

Freeze-dried powder (500 mg) was suspended in 60% MeOH/water solution (20 mL) and boiled for 5 min. The powder was filtered and freeze-dried again.

### 2.8. Decoction in n-Hexane

Freeze-dried powder (500 mg) was suspended in n-hexane (40 mL) and boiled for 6 h. Then the powder was filtered and freeze-dried again. 

### 2.9. Phytochemical Profile

To provide first insight on the phytochemical content of the tested pepper samples, we determined total bioactive compounds (total phenolic (TPC) and total flavonoid (TFC)) in the extracts [14]. Standard compounds were used for evaluating the results. Gallic acid and rutin were used for TPC and TFC, respectively. Stock solutions preparation, analytical settings and quantitative analyses were performed and applied following previously documented literature data [12,13,15].

### 2.10. Fatty Acids, Methyl Esters (FAMEs) Preparation and Gas Chromatographic Analysis

Fatty acid composition of the n-hexane extracts from pepper samples were determined by GC- flame ionization detector (FID, Hewlett Packard, Palo Alto, CA, USA). Firstly, the fatty acids were converted to methyl esters by using BF_3_ (14%, methanolic) [16]. The chromatographic analysis was performed by using a HP Agilent 6890 N (Palo Alto, CA, USA), HP-88 capillary column (100 m, 0.25 mm i.d. and 0.2 μm), and a flame ionization detector (FID) was used to separate and identify these fatty acids [17]. Chromatographic conditions were given in our earlier paper [18].

### 2.11. Antioxidant Properties 

Phosphomolybdenum, metal chelating, cupric reducing antioxidant capacity (CUPRAC), ferric reducing antioxidant power (FRAP), 2,2ʹ-azino-bis(3-ethylbenzothiazoline-6-sulfonic acid) (ABTS) and 2,2-diphenyl-1-picrylhydrazyl (DPPH) assays were performed following Savran et al. [19], using **s**tandard equivalents (Trolox and EDTA) to determine antioxidant ability.

### 2.12. Enzyme Inhibitory Assays

The possible enzymatic inhibitory activities were revealed following standard in vitro bioassays against acetylcholinesterase (AChE), butyrylcholinesterase (BChE) (by Ellman’s method), α-amylase, α-glucosidase and tyrosinase. Results are expressed as standard equivalents of acarbose for α-amylase and α-glucosidase; kojic acid for tyrosinase; galantamine for cholinesterases [20].

### 2.13. Cytotoxic Activity Test

3-(4,5-dimethylthiazol-2-yl)-2,5-diphenyltetrazolium bromide (MTT)-based cell viability analysis was assessed to evaluate the cytotoxic activity of extracts against prostate cancer line 3 (PC3). PC3 cell line was obtained from Department of Biochemistry, Faculty of Science, Selcuk University (Konya, Turkey). Pepper extracts were dissolved in 1× phosphate buffered saline (PBS). Prostate cancer (PC3) cells were cultured in high glucose Dulbecco’s Modified Eagle Medium (DMEM), including 10% fetal bovine serum (FBS), penicillin/streptomycin and 1% non-essential amino acid at 37 °C in a 5% CO_2_ humidified incubator. Passage of cells was performed when they reached 80% confluence. Ten thousand cells were seeded in 96-well plates and allowed to cling on the well for 24 h. Cells were treated with various aliquots (62.5 µg/mL, 125 µg/mL, 250 µg/mL, 500 µg/mL and 1000 µg/mL) of extracts (hot pepper and sweet pepper) for 24 h. Control cells were treated with PBS. Cytotoxicity analysis was carried out as mentioned before [21]. After treatment duration was completed, the medium of cells was changed with MTT and incubated for 4 h. Formazan crystals of cells were liquefied in 3% SDS and HCl/isopropanol solution and homogenized. After that, absorbance of samples was measured under a microplate reader (Euroclone Spa, Pero, Milano, Italy) at 570 nm. After percentages of cell survival rates were calculated, half-maximal inhibitory concentrations of extracts were determined according to log (inhibitor) vs. normalized response–variable slope analysis function with the aid of GraphPad Prism 7 software (GraphPad Software, San Diego, CA, USA). 

### 2.14. In Vivo Anti-Inflammatory Assay 

The in vivo anti-inflammatory assay was performed following our previously reported literature [22]**.** The research protocol was approved by the Service for Biotechnology and Animal Welfare of the Italian National Institute of Health and authorized by the Italian Ministry of Health (authorization number, 198/2013-B) according to Legislative Decree 116/92 and 26/14, which implemented the European Directive 2010/63/UE on laboratory animal protection in Italy. 

### 2.15. Statistical Analysis 

GraphPad Prism version 5.01 for Windows (GraphPad Software, San Diego, CA, USA) was used for the statistical analysis together with means ± standard deviations (SDs) for each experimental group. Firstly, the results were tested by the Shapiro–Wilk test (*p* < 0.05) for normality. Some parameters (total phenolic content, DPPH, ABTS, CUPRAC, metal chelating, phosphomolybdenum, and AChE, tyrosinase, amylase and glucosidase inhibitions) did not have normal distribution. For these parameters, we used Kruskal–Wallis for evaluating differences in the extracts (*p* < 0.05). For other parameters (total flavonoid, FRAP and BChE inhibition), we used one-way analysis of variance (ANOVA) (*p* < 0.05). For in vitro pharmacological data, we used ANOVA followed by a Newman–Keuls comparison multiple test to determine the concentration–response relationship. Data from in vivo experiments were analyzed using ANOVA followed by Dunnett’s multiple comparisons test, in which statistical significance was set at *p* < 0.05

Total bioactive compounds (TPC and TFC) and detected biological activities were statistically analyzed by calculating the Pearson correlation coefficient. Obtained results were submitted to supervised partial least squares discriminant analysis (PLS-DA) in an attempt to discriminate pepper samples. Variable importance in projection (VIP) was used to determine the importance of each biological activity in pepper separation. Biological activities with VIP score > 1 were considered to have the highest discrimination potential. Finally, a dotplot was achieved, to view the effect of extraction solvents on biological activities of each particle. R v 3.6.1 statistical software (The R Foundation, Middle East Technical University, Northern Cyprus Campus, Mersin, Turkey) was employed for the analysis. 

## 3. Results and Discussion

### 3.1. Phytochemical Composition

The total phenolic and flavonoid contents were determined by Folin–Ciocalteu and AlCl_3_ methods, respectively (Table 1). Hot and sweet peppers did not show high concentration of these bioactive compounds; however, the hot pepper extracts exhibited the highest content of phenolic compounds compared to sweet pepper. The Soxhlet extraction of sweet pepper exhibited the highest content of flavonoid. Phenolic components were shown performing a validated HPLC protocol to determine the presence and amount of secondary metabolites (Table 2) [23,24]. The applied method, validated on another plant material, after a check for the absence of interferences and a check of the analytical signal response (that allows consideration of the previously validated figure of merits), was directly applied to the herein reported extracts.

Regarding the total phenolic content in the hot pepper extracts, the highest level was noted in decoction, followed by microwave and Soxhlet. As for sweet pepper extracts, Soxhlet reported the highest amount of total phenolics.

In all preparations, catechin was detected in moderate amounts. Other biologically active compounds such as 3-OH-benzoic acid, naringenin and carvacrol were present in low concentrations. In terms of the total content in hot pepper microwave and Soxhlet extractions, the major compound was catechin, while in the decoction with n-hexane extraction technique, carvacrol, naringenin and 3-OH-benzoic acid were predominant. For the sweet pepper, the microwave and the decoction extraction techniques achieved better recovery of these metabolites. 

### 3.2. Antioxidant Properties

All extracts of *C. annum* were tested in vitro, by performing free radical scavenging (DPPH and ABTS) and reducing power (CUPRAC and FRAP), and phosphomolybdenum and metal chelating assays. For the hot pepper, the decoction extracts exhibited significant abilities in all the antioxidant assays. Modest values were achieved by the free radical inhibitory activities of the decoction extract (22.13 mg TE/g extract for DPPH and 52.00 mg Trolox equivalent (TE)/g extract for ABTS) while those of the decoction n-hexane extract for DPPH and ABTS (respectively, 2.01 and 18.17 mg TE/g) were the lowest. 

**Figure 2:**
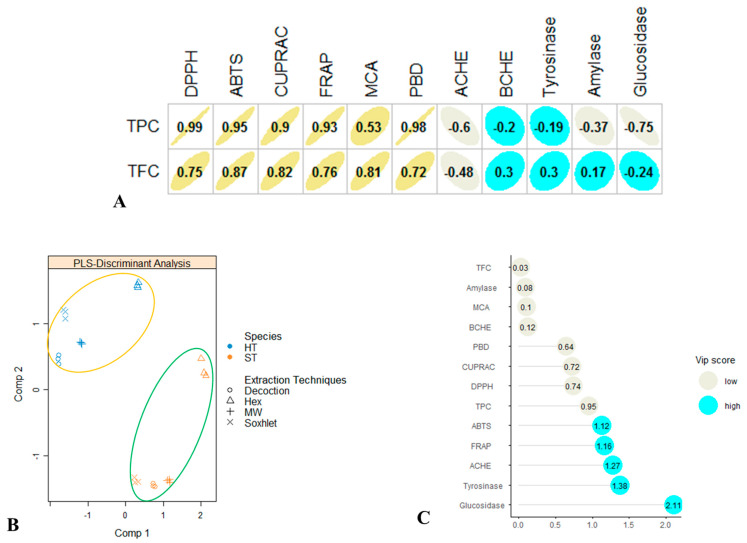
Multivariate analysis. **A**: Relationship among total phenolic compound (TPC), total flavonoid compound (TFC) and biological activities (Pearson correlation coefficient). **B**: A sample plot from supervised partial least squared discriminant analysis on biological activities of pepper extracts (HT: hot pepper; ST: sweet pepper). **C**: The most discriminant biological activities were identified through variable importance in projection (VIP) score calculation.

The reducing power of hot pepper by CUPRAC can be ranked as follows: decoction > Soxhlet > MW > hexane (Table 3). This is probably due to the best concentration of phenolic compounds in decoction extract [25]. This hypothesis is also confirmed by Pearson correlation coefficient values reported in Figure 2, and we observed high correlation values between total phenolic content and antioxidant properties (*R* = 0.99 for DPPH, *R* = 0.95 for ABTS, *R* = 0.9 for CUPRAC and *R* = 0.93 for FRAP). Furthermore, recent studies reported a linear correlation between the phenolic amounts and the antioxidant activities of extracts [25,26].

The highest activity in the phosphomolybdenum assay was recorded from hot pepper decoction, and the lowest from the hot pepper n-hexane decoction. These results confirmed that the major polyphenol content of hot pepper decoction increases antioxidant activities. The highest metal chelating abilities in hot pepper samples were found in Soxhlet extraction with 19.25 mg EDTA equivalent (EDTAE)/g, probably due to the amount of non-phenolic compound. The best results in terms of bioactive compound quantity and their relative antioxidant abilities were obtained from hot pepper samples. 

### 3.3. Evaluation of Enzyme Inhibition 

Enzyme inhibition is one of the most efficacious approaches applied to treat serious diseases such as Alzheimer’s disease (AD) and diabetes mellitus (DM) [27]. According to this, we evaluated the inhibitory effects of sweet and hot pepper extracts against different enzymes involved in these pathologies. Table 4 shows modest inhibition values against cholinesterases, important therapeutic targets for modulating neurotransmission in Alzheimer’s disease. The AChE inhibition activity of n-hexane was 1.87 mg galantamine equivalent (GALAE)/g for hot pepper and 2.25 mg GALAE/g for sweet pepper, which was the highest value among all extracts. In contrast, the microwave extracts exhibited the weakest AChE inhibitory activity (0.29 mg GALAE/g extract for hot pepper and not active for the sweet pepper). In the BChE inhibition, modest values were achieved by the Soxhlet sweet pepper extract with 5.99 mg GALAE/g and by the n-hexane preparation for the hot species. These results may be justified by phytochemical composition, considering that diverse bioactive compounds could act on different binding sites. In this study, we also described the inhibition of tyrosinase enzyme for all the extracts [28].

According to this, the hot pepper microwave extract shows good activity with 59.60 mg kojic acid equivalent (KAE)/g, followed by the Soxhlet extract. Regarding the sweet pepper inhibition of the same enzyme, the best value was registered for microwave and Soxhlet extracts. This could be related to the extractive techniques which provided for high content of antityrosinase agents such as catechin. α-Amylase inhibition abilities of sweet and hot types can be ranked as follows: microwave > Soxhlet > n-hexane > decoction. Hexane extracts exhibited modest values in glucosidase inhibition, followed by Soxhlet and microwave for sweet and hot pepper.

**Figure 3:**
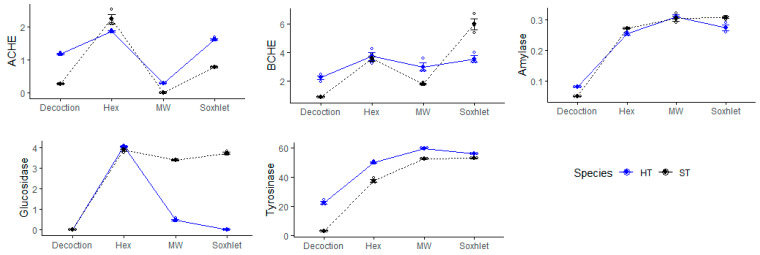

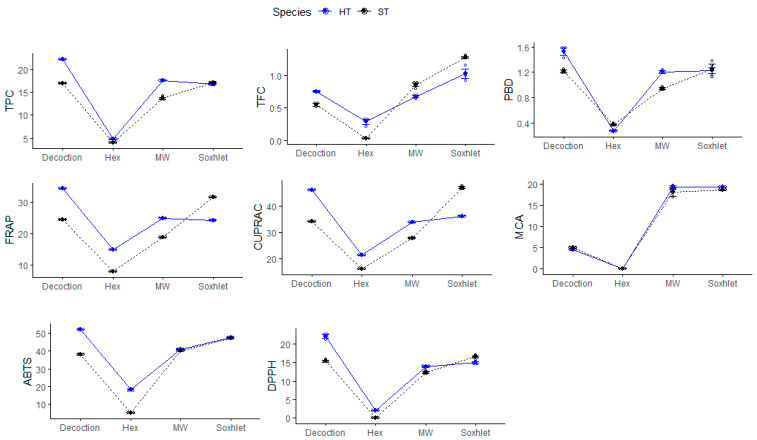
Dotplot for exploring the effect of extraction techniques on biological activities of pepper samples (HT: hot pepper; ST: sweet pepper).

To provide more insights on the tested pepper samples, we performed multivariate analysis. PLS-DA is one of the most suitable methods for this purpose. Figure 2 indicates that the pepper samples are clearly different in terms of total bioactive compounds and biological activities (antioxidant and enzyme inhibitory effects) (Figure 2B). We determined which parameters provided for the separation; thus, VIP scores were calculated. ABTS, FRAP, AChE and tyrosinase inhibition were higher than 1 and thus these parameters were the most important for pepper discrimination (Figure 2C). Dotplot also indicated that the extraction methods influenced the total bioactive compounds and biological activities (Figure 3).

### 3.4. Fatty Acid Composition

The fatty acid composition of *C*. *annuum* oil was assessed by GC-FID technique (Table 5). Linoleic acid (C 18:2 ω6) and polyunsaturated fatty acid (PUFA) were found to be the major fatty acids in 37.19% and 33.73% of sweet and hot pepper samples, respectively. Other predominant fatty acids observed in sweet pepper samples were palmitic acid (C 16, 23.40%), stearic acid (C 18, 12.00%) and γ-linoleic acid (C 18:3 ω6, 8.52%). For the hot pepper, palmitic acid (29.18%), oleic acid (16.69%) and γ-linoleic acid (8.14%) were the most frequently found fatty acids. 

The essential fatty acids content was more than 45% of total fatty acids for sweet pepper samples and more than 41% for hot pepper samples. Oleic acid (C 18: 1 ω9) was the major monounsaturated fatty acid component (MUFAs), with contents ranging from 8.44% in the sweet pepper oils to 16.69% in hot pepper oils. Total PUFA content was higher than the total saturated fatty acid content (SFA) in both types of oils. In light of these results, we can suppose that the sweet pepper is a source of essential fatty acids while the hot pepper is rich in unsaturated fatty acids. 

### 3.5. In Vitro Cell Assay

One of the most common diseases in developed countries is prostate cancer (PC), which is strongly associated with risk factors such as tobacco use, obesity and physical inactivity [29]. Infections, dietary factors and hormonal changes are etiological factors that can contribute to prostatic inflammation. In particular, dietary mutagens such as heterocyclic amines (HCAs), produced when meat is cooked at high temperature, presents some correlations with cancers of various organs [30,31].

**Figure 4:**
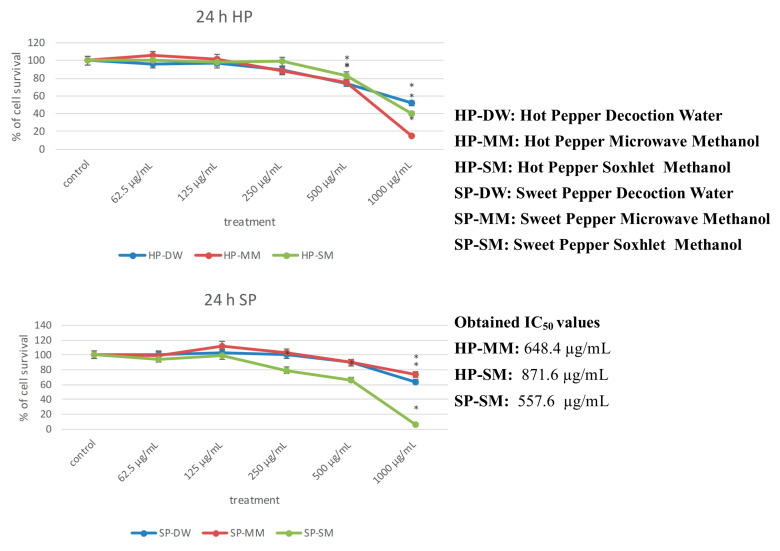
Inhibition values of cell growth with hot and sweet pepper extracts (* *p* < 0.05).

**Figure 5:**
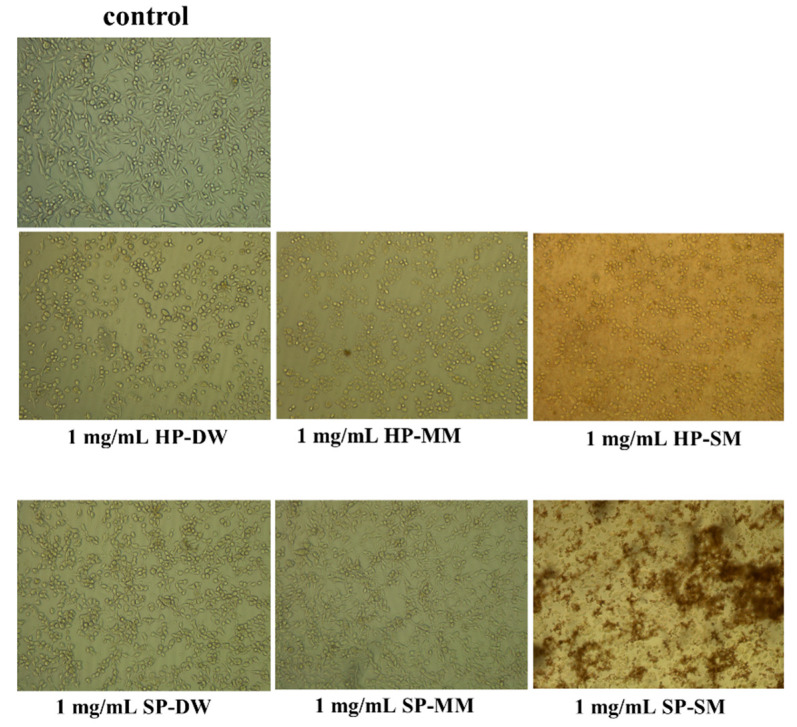
Effect of hot and sweet pepper extracts on prostate cancer cell line 3 (PC3) cells under inverted microscope using 10× objective.

Recent studies have examined the ability of capsaicin to trigger autophagy in prostate cancer androgen-sensitive cells and the role of autophagy in capsaicin-induced cytotoxicity [32,33]. Based on these studies, we tested the cell growth inhibition of each extract. According to the results of the enzyme inhibition tests, the microwave for hot pepper and Soxhlet for sweet pepper were the best methods of extractions. In cells tested with hot pepper, the best inhibition values of cell growth were recorded by the microwave and Soxhlet extracts. After treatment with 1000 mg/mL of microwave extract, the percentage of cells that survived was about 20%. After treatment with Soxhlet extracts, the percentage of surviving cells was 40%, while the decoction extract did not provide significant data (Figure 4). In the sweet pepper samples, cells that survived after treatment with 1000 mg/mL were less than 20% in the Soxhlet extract (IC_50_ 557.6 μg/mL). The Soxhlet extract of sweet pepper exhibited the best value of inhibition. The microwave and decoction extracts of sweet pepper did not exert a significant effect on cellular growth (Figure 5). This finding could be a starting point for new studies on cancer activity of different pepper varieties with different content of capsaicin.

### 3.6. In Vivo Anti-Inflammatory Assay

Anti-inflammatory properties of diverse plants such as green tea, soy and tomato products have been associated with a decrease in human prostate cancer risk [34,35].

**Figure 6:**
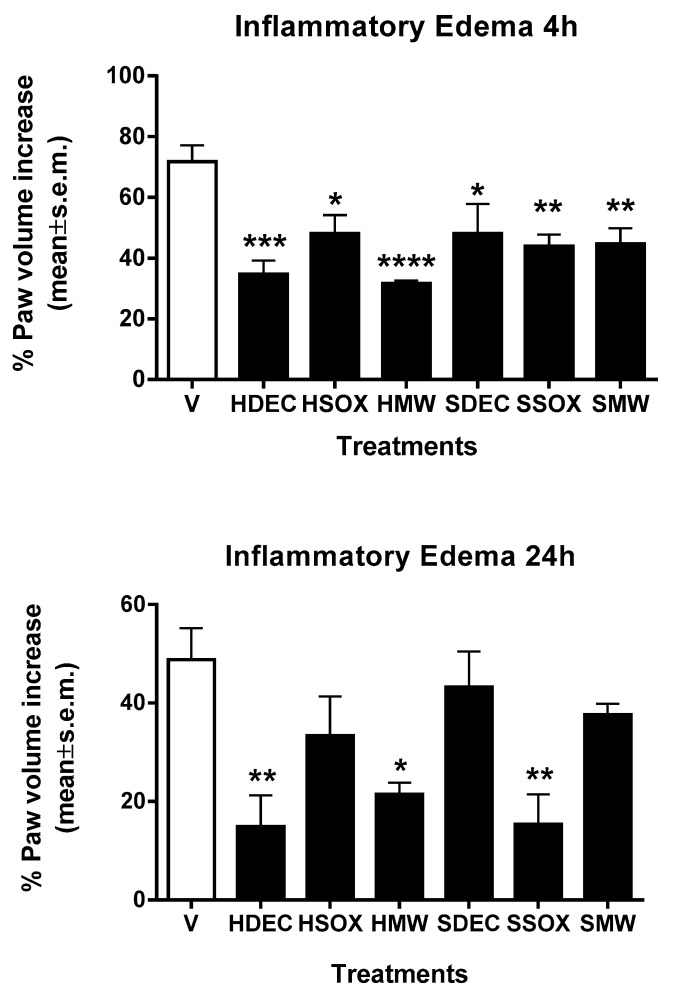
Effect of hot pepper decoction (HDEC), hot pepper Soxhlet (HSOX), hot pepper microwave (HMW), sweet pepper decoction (SDEC), sweet pepper Soxhlet (SSOX) and sweet pepper microwave (SMW) on paw edema formation in mice. Extracts were administered in the mice paw 30 min before zymosan at a dose of 100 μg/paw. * *p* < 0.05, ** *p* < 0.01, *** *p* < 0.001 and **** *p* < 0.0001 vs. V (vehicle-treated animals). *n* = 7.

Zymosan-induced paw inflammation in laboratory animals is a well-established assay to investigate edema responses to anti-inflammatory drugs [36]. The results of this assay are reported in Figure 6, and showed that the extracts induced anti-inflammatory effects inhibiting edema formation; the best inhibition values were achieved by the decoction and microwave extracts of hot pepper, observed both at 4 and 24 h after zymosan administration. Other extracts also achieved good values of edema inhibition. These data are significant since the literature has documented a strong correlation between inflammation and prostate cancer [37,38,39,40]. 

## 4. Conclusions

Among claimed functional foods, sweet and hot peppers are interesting for their nutritional and organoleptic values but also as natural sources of carotenoids and polyphenols. In this work, we performed a preliminary analysis of the total flavonoid/phenolic contents, antioxidant, anti-inflammatory activities and enzyme inhibition properties of four different extracts (Soxhlet extract, microwave extract, two decoction extracts) of sweet and hot peppers from Altino (Chieti, Italy). The best polyphenolic content was detected in the Soxhlet extract of the sweet pepper, which was characterized by a significant antioxidant property and high enzyme inhibition activity. Furthermore, this extract exhibits good value of antityrosinase activity, which could open new studies regarding the inhibitory ability for different varieties of peppers. Microwave extract of hot pepper exhibited the best tyrosinase inhibition effect while decoction extract was the best technique in terms of polyphenol content and antioxidant activities. The content of polyunsaturated and essential fatty acids was high in sweet peppers, where this latter component was predominant. Noteworthy high intake of long chain anti-inflammatory n-3 PUFA (omega 3) has been recently associated to decrease risk in prostate cancer [34]. In cells treated with hot pepper, the best inhibition values of cell growth were recorded by the microwave and Soxhlet extracts; in fact, after treatment with 1000 mg/mL, microwave extract provided for about 20% survived cells, while after treatment with Soxhlet extracts, the percentage of survived cells was equal to 40%. In vivo anti-inflammatory assay was performed, revealing high anti-inflammatory effects for all the extracts through edema formation. The two latter data, taken together, could explain the protective activity reported by the literature on human prostatic hyperplasia [41,42]. Overall, the present results highlight the functional role of sweet and hot peppers from Altino and their different extraction techniques, suggesting a possible use as nutraceutical sources. 

## Figures and Tables

**Table 1:** Total phenolic and flavonoid contents in the tested extracts^*^.

Samples	Methods	Total Phenolic Content (mg GAE/g)^x^	Total Flavonoid Content (mg RE/g) ^y^
Hot pepper	Decoction	22.19 ± 0.14^a^	0.75 ± 0.01^cd^
	Soxhlet	16.82 ± 0.38^bc^	1.03 ± 0.12^b^
	Microwave	17.65 ± 0.32^ab^	0.67 ± 0.03^de^
	Hexane	4.71 ± 0.08^c^	0.28 ± 0.06^f^
Sweet pepper	Decoction	17.00 ± 0.14^abc^	0.55 ± 0.04^e^
	Soxhlet	17.09 ± 0.28^ab^	1.28 ± 0.03^a^
	Microwave	13.69 ± 0.47^bc^	0.85 ± 0.05^c^
	Hexane	4.00 ± 0.04^c^	0.03 ± 0.01^g^

* Values expressed as means ± SD of three parallel measurements. GAE: gallic acid equivalent; RE: rutin equivalent. In same column, different superscripts (a–g) indicate significant differences (between means) in the tested extracts (*p* < 0.05). ^x^ Statistical evaluation by Kruskal–Wallis test. ^y^ Statistical evaluation by ANOVA test.

**Table 2:** Quantitative analysis on hot pepper and sweet pepper by means of the published method.

Concentration								
Standard Compounds	Hot Pepper				Sweet Pepper			
	Microwave	Soxhlet	Decoction	Decoction *n*-Hexane	Microwave	Soxhlet	Decoction	Decoction *n*-Hexane
Gallic acid						0.21 ± 0.02		
Catechin	3.49 ± 0.14	2.47 ± 0.13	0.76 ± 0.08	0.32 ± 0.03	5.22 ± 0.17	0.56 ± 0.05	5.09 ± 0.23	
Chlorogenic acid								
*p*-OH benzoic acid								
Vanillic acid			BLD	BLD			BLD	
Epicatechin								
Syringic acid			0.22 ± 0.02					
3-OH benzoic acid	0.58 ± 0.05			1.20 ± 0.09		0.34 ± 0.03		
3-OH-4-MeO benzaldehide								
*p*-Coumaric acid								
Rutin		BLQ					BLD	
Sinapinic acid						BLD	BLD	
*t*-Ferulic acid	BLD					0.40 ± 0.03		
Naringin								
2.3-diMeO benzoic acid								
Benzoic acid							0.25 ± 0.02	
*o*-Coumaric acid								
Quercetin dihydrate								
Harpagoside								
*t*-Cinnamic acid								
Naringenin	1.64 ± 0.18	1.31 ± 0.13		1.25 ± 0.11			1.28 ± 0.12	
Carvacrol		0.28 ± 0.03	0.47 ± 0.05	2.50 ± 0.22	0.31 ± 0.03			
Total (µg/mL)	5.71	4.07	1.45	5.27	5.53	1.50	6.63	0.00
Total (µg/mg) (solid:liquid ratio 1:1. *w:v*)	5.71	4.07	1.45	5.27	5.53	1.50	6.63	0.00

**Table 3:** Antioxidant properties of the tested extracts.

Samples	Methods	DPPH (mg TE/g)^x^	ABTS (mg TE/g)^x^	CUPRAC (mg TE/g)^x^	FRAP (mg TE/g)^y^	PHBD (mmol TE/g)^x^	Chelating Activity (mg EDTAE/g) ^y^
Hot pepper	Decoction	22.13 ± 0.86^a^	52.00 ± 0.12^a^	46.23 ± 0.31^ab^	34.38 ± 0.25^a^	1.5 3± 0.09^a^	4.47 ± 0.17^bc^
	Soxhlet	15.08 ± 0.52^abc^	47.62 ± 0.33^ab^	36.26 ± 0.42^abc^	24.18 ± 0.22^d^	1.23 ± 0.08^abc^	19.25 ± 0.03^a^
	Microwave	13.91 ± 0.35^bcd^	40.98 ± 0.52^abcd^	33.83 ± 0.18^bcde^	24.85 ± 0.38^c^	1.21 ± 0.04^bcd^	19.15 ± 0.37^a^
	Hexan	2.01 ± 0.08^cd^	18.17 ± 0.90^de^	21.35 ± 0.28^de^	14.84 ± 0.05^f^	0.27 ± 0.02^d^	NA
Sweet pepper	Decoction	15.44 ± 0.55^ab^	38.01 ± 0.56^cde^	34.14 ± 0.10^abcd^	24.42 ± 0.24	1.21 ± 0.04^bcd^	4.93 ± 0.16^bc^
	Soxhlet	16.65 ± 0.47^ab^	47.08 ± 0.16^abc^	47.15 ± 0.62^a^	31.60 ± 0.21^b^	1.25 ± 0.13^ab^	18.49 ± 0.04^ab^
	Microwave	12.37 ± 0.46^bcd^	40.20 ± 0.51^bcde^	27.95 ± 0.24^cde^	18.74 ± 0.26^e^	0.94 ± 0.03^bcd^	18.05 ± 1.05^abc^
	Hexan	NA	5.17 ± 0.16^e^	16.21 ± 0.11^e^	7.81 ± 0.07^g^	0.38 ± 0.03^cd^	NA

Values expressed as means ± SD of three parallel measurements; PHBD: phosphomolybdenum assay; TE: Trolox equivalent; EDTAE: EDTA equivalent; NA: not active. In same column, different superscripts (a–g) indicate significant differences in the tested extracts (*p* < 0.05). ^x^ Statistical evaluation by Kruskal–Wallis test. ^y^ Statistical evaluation by ANOVA test.

**Table 4:** Enzyme inhibitory properties of the tested extracts.

Samples	Methods	AChE Inhibition (mg GALAE/g)^x^	BChE Inhibition (mg GALAE/g)^y^	Tyrosinase Inhibition (mg KAE/g)^x^	Amylase Inhibition (mmol ACAE/g)^x^	Glucosidase Inhibition (mmol ACAE/g)
Hot pepper	Decoction	1.18 ± 0.05^abc^	2.21 ± 0.26	22.60 ± 1.95^cd^	0.08 ± 0.01^b^	NA
	Soxhlet	1.63 ± 0.05^ab^	3.54 ± 0.39^b^	55.83 ± 0.26^ab^	0.27 ± 0.01^ab^	NA
	Microwave	0.29 ± 0.01^bcd^	2.98 ± 0.53^bc^	59.60 ± 0.28^a^	0.31 ± 0.01^a^	0.48 ± 0.07^abc^
	Hexan	1.87 ± 0.05^ab^	3.72 ± 0.51^b^	49.94 ± 1.08^bcd^	0.26 ± 0.01^b^	4.05 ± 0.04^a^
Sweet pepper	Decoction	0.28 ± 0.02^cd^	0.82 ± 0.09^e^	3.24 ± 0.66^d^	0.05 ± 0.01^b^	NA
	Soxhlet	0.79 ± 0.04^bcd^	5.99 ± 0.67^a^	52.71 ± 0.44^abc^	0.30 ± 0.01^a^	3.72 ± 0.07^a^
	Microwave	NA	1.78 ± 0.11^de^	52.48 ± 0.94^ab^	0.31 ± 0.01^a^	3.37 ± 0.01^ab^
	Hexan	2.25 ± 0.24^a^	3.55 ± 0.30^b^	37.40 ± 1.82^bcd^	0.27 ± 0.01^ab^	3.87 ± 0.11^a^

Values expressed as means ± SD of three parallel measurements. AChE: acetylcholinesterase; BChE: butyrylcholinesterase; GALAE: galantamine equivalent; KAE: kojic acid equivalent; ACAE: acarbose equivalent. NA: not active. In same column, different superscripts (a–e) indicate significant differences in the tested extracts (*p* < 0.05). ^x^Statistical evaluation by Kruskal–Wallis test. ^y^Statistical evaluation by ANOVA test.

**Table 5:** Fatty acids composition for hot and sweet peppers from Altino (%).

Fatty Acids	Sweet Pepper	Hot Pepper
C 12:0	2.69 ± 0.04 *	0.96 ± 0.04
C 14:0	5.85 ± 0.08	3.07 ± 0.05
C 15:0	0.09 ± 0.01	0.08 ± 0.01
C 16:0	23.40 ± 0.39	29.18 ± 0.25
C 17:0	0.05 ± 0.01	0.16 ± 0.03
C 18:0	12.00 ± 0.23	6.86 ± 0.09
ΣSFA	44.07 ± 0.11	40.30 ± 0.22
C 14:1 ω5	0.11 ± 0.01	0.11 ± 0.01
C 15:1 ω5	0.24 ± 0.01	0.05 ± 0.02
C 16:1 ω7	0.85 ± 0.02	0.57 ± 0.03
C 17:1 ω8	0.23 ± 0.01	0.05 ± 0.01
C 18:1 n9	8.44 ± 0.13	16.69 ± 0.23
ΣMUFA	9.85 ± 0.11	17.46 ± 0.19
C 18:2 ω6	37.19 ± 0.19	33.73 ± 0.06
C 18:3 ω6	0.38 ± 0.03	0.38 ± 0.01
C 18:3 ω3	8.52 ± 0.46	8.14 ± 0.07
ΣPUFA	46.08 ± 0.24	42.25 ± 0.03
ΣUFA	55.93 ± 0.13	59.71 ± 0.22
ΣEFA	45.70 ± 0.27	41.87 ± 0.01

* Values are reported as mean ± SD. SFA: saturated fatty acids; MUFA: monounsaturated fatty acids; PUFA: polyunsaturated fatty acids; UFA: unsaturated fatty acids; EFA: essential fatty acids.
